# Insight into the transient inactivation effect on Au/TiO_2_ catalyst by in-situ DRIFT and UV–vis spectroscopy

**DOI:** 10.1038/s41467-022-33187-y

**Published:** 2022-09-17

**Authors:** Xianwei Wang, Arnulf Rosspeintner, Abolfazl Ziarati, Jiangtao Zhao, Thomas Bürgi

**Affiliations:** Department of Physical Chemistry, University of Geneva, 1211 Geneva 4, Switzerland

**Keywords:** Heterogeneous catalysis, Infrared spectroscopy

## Abstract

Au catalysts have drawn broad attention for catalytic CO oxidation. However, a molecular-level understanding of the reaction mechanism on a fast time-resolved scale is still lacking. Herein, we apply in situ DRIFTS and UV-Vis spectroscopy to monitor the rapid dynamic changes during CO oxidation over Au/TiO_2_. A pronounced transient inactivation effect likely due to a structural change of Au/TiO_2_ induced by the reactants (CO and O_2_) is observed at the beginning of the reaction. The transient inactivation effect is affected by the ratio of CO and O_2_ concentrations. More importantly, during the unstable state, the electronic properties of the Au particles change, as indicated by the shift of the CO stretching vibration. UV-Vis spectroscopy corroborates the structure change of Au/TiO_2_ surface induced by the reactants, which leads to a weakening of the Au catalyst’s ability to be oxidized (less O_2_ adsorption), resulting in the transient inactivation effect.

## Abstract (web summary)

A molecular-level understanding of the Au-catalyzed CO oxidation on a fast time-resolved scale is still lacking. Here the authors monitor the rapid dynamic changes during CO oxidation over Au/TiO2 using in situ DRIFTS and UV-Vis spectroscopy, and reveal that the catalyst undergoes a surprising structural change at the beginning of the reaction.

## Introduction

Catalysis, especially heterogeneous catalysis, is of vital importance to global development, as it plays a prominent role in the current chemical industry and for the development of sustainable technologies in areas like the production of fine chemicals, oil refining, environmental issues, and clean fuels^1,2^. Heterogeneous catalysts proceed by adsorption of reactant molecules to increase the reactant concentration on the surface^3^ and by guiding the reacting molecules toward alternative reaction pathways. Heterogeneous catalysts have huge advantages compared to homogeneous ones concerning the separation of the catalyst from the products^2^. Among the heterogeneous catalysts, supported finely dispersed metal particles or single atoms are the most widespread form^1^. The high catalytic activity of Au, a precious metal, was initially reported by Haruta and Hutchings^4–7^. It was then soon realized that highly dispersed Au nanoparticles (NPs) exhibit exceptional catalytic activity for many reactions, including CO oxidation.

The CO oxidation reaction is one of the most widely studied processes in the area of Au catalysis. It is also vital to our understanding of a range of related reactions, such as the preferential oxidation of CO in the presence of H_2_ (PROX) and the water–gas shift (WGS) reaction^8^. Since the discovery that Au NPs with diameters of 3–5 nm exhibited surprisingly high catalytic activity for CO oxidation at low temperature, tremendous efforts using various kinds of sophisticated techniques have been made to understand the reaction mechanism of supported gold catalysts for CO oxidation^9–14^. From these studies it emerges that the important factors for CO oxidation on Au catalysts include: (i) the type of support, (ii) the structure of the contact area between the Au particles and the support, (iii) the size of Au particles, and (iv) the presence of moisture^14^. For the identification and tracking of surface intermediates, operando and in situ methods are of central importance^15–20^. However, most of these studies focused on a time scale of minutes to hours. Only a few studies focus on the mechanism and processes taking place on a fast time-scale of CO oxidation over gold catalysts, whereas changes in the catalyst and composition of the adsorbate layer can occur very fast. In fact, some intermediates can be generated or dynamic processes can happen at the time-scale of nanoseconds to seconds^19–22^. There is no doubt that some interesting short-lived intermediates or fast processes may be obscured by low time-resolution experiments. Also, during the initial stage of the reaction, before reaching a steady state, important phenomena may take place the unraveling of which may contribute to a better understanding of the catalyst. Therefore, obtaining more details of the reaction kinetics through high time-resolution methods can further enrich the knowledge about the reaction mechanism.

Infrared spectroscopy, especially Diffuse Reflectance Infrared Fourier Transform Spectroscopy (DRIFTS), is one of the most important techniques for in situ studies of catalytic solids under reaction conditions^23^. The development of DRIFTS combined with modulation excitation spectroscopy (MES) has important advantages for studies of the transient period following the (periodic) switching of one or several reaction parameters (such as reactant concentration). The interactions at catalytic solid–gas interfaces can be detected with much higher sensitivity compared to conventional DRIFT spectroscopy due to the phase-sensitive detection (PSD) of periodically varying signals and the high selectivity for species, which are affected by the stimulation^24^.

In this work, we study the dynamic process of CO oxidation on Au/TiO_2_ surface at a fast time-resolved scale in a small-volume DRIFT and UV-vis microreactor. The design of the cell allows rapid exchange of the reactant mixture and detecting IR signals originating from diffuse reflectance at the catalyst surface thus enabling us to study the dynamic processes on the catalyst surface taking place during a very short period at the beginning of the reaction. With the help of this advanced cell, a transient inactivation period is discovered during the CO oxidation on the Au/TiO_2_ surface. During this period the electronic properties of the catalyst particles change rapidly, as indicated by the frequency of the CO stretching vibration. Optical spectroscopy in the UV-vis-NIR region indicates a reversible structure change of the catalyst.

## Results and discussion

### FTIR study of CO adsorption and CO oxidation

**Fig. 1:**
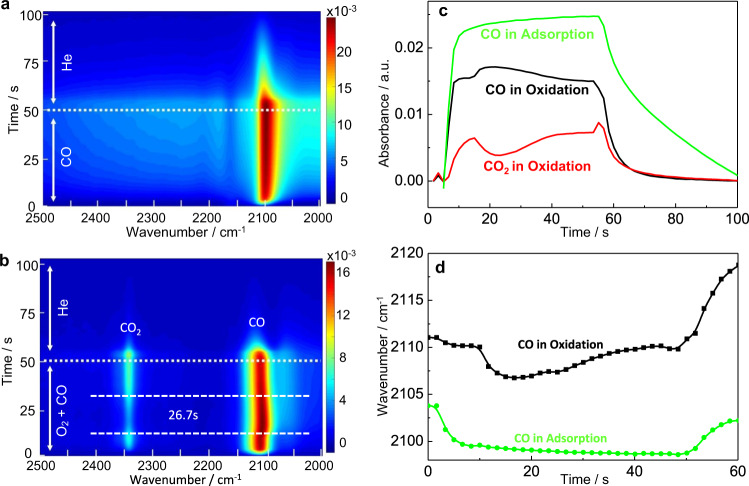
FTIR spectra of CO adsorption and CO oxidation on Au/TiO_2_ at 25 °C. **a** CO adsorption; **b** CO oxidation. Plots of CO and CO_2_ band intensity against time (**c**) and plot of the CO stretching band maximum against time (**d**) for an experiment at 25 °C. Experimental conditions: Flow rate is 30 mL min^−1^; modulation period is 100 s; number of spectra in one cycle is 60. For CO adsorption, during the first half-cycle 1% CO in He was flowed through the cell, then the feed gas was switched to He for the second half-cycle. For CO oxidation, during the first half-cycle 1% CO and 1% O_2_ in He was flowed through the cell, then the feed gas was switched to He for the second half-cycle.

CO adsorption on Au/TiO_2_ catalyst was carried out first. As shown in Fig. 1a, c and Supplementary Fig. 1, after introducing CO gas, a sharp peak at around 2100 cm^−1^ due to the CO adsorption on Au rapidly increased initially (in about 6 s) followed by a much slower growth of the CO signal until the gas changed to He. Then the adsorbed CO gradually desorbed from the catalyst surface. In addition to this band, a broadband infrared (BB-IR) signal between 1500 and 3000 cm^−1^ was also observed. A similar BB-IR signal was observed before and assigned to a decrease in transmission through the powder sample when CO is adsorbed on Au/TiO_2_ catalyst^25^. It is related to the reversible, partial reduction of the TiO_2_ at the Au-TiO_2_ interface^25^. At higher temperatures, such as 150 °C (Supplementary Fig. 2), the intensity of this broadband was enhanced. Note that there is no band visible in the spectra due to CO gas and the absorbance of CO adsorbed on Au could reach up to 0.02 a.u. The high intensity of the adsorbed species and the absent (or low) gas-phase signal demonstrates the potential of the used low-volume cell for in situ studies of heterogeneous catalytic reactions. In addition, independent of the temperature, the intensity of adsorbed CO peak was smooth and stable, indicating that the system has a high stability for the rapid gas exchange of reactants. Quick and complete exchange of the reactant in the cell is important to restart the reaction over the catalyst surface in modulation experiments. Modulation experiments using CO_2_ as a test gas (30 mL min^–1^) showed that the gas in the cell could be completely exchanged within less than 1 s (Supplementary Fig. 3).

Next, we performed CO oxidation experiments on the Au/TiO_2_ catalyst by admitting a mixture of carbon monoxide and oxygen. To our surprise, the observed signals of adsorbed CO and gas-phase CO_2_ did not show a smooth transition to a steady state. In fact, a transient deactivation of the CO oxidation was observed as indicated by a decrease of the CO_2_ signal. As shown in Fig. 1b, c, after introducing CO and O_2_ gas, a new band at 2343 cm^−1^ assigned to the product (CO_2_) emerged showing that CO oxidation occurred^26,27^. Also, some bands between 1700 and 1200 cm^−1^ assigned to carbonate species emerged in the spectra (Supplementary Figs. 4a and 5). In the initial stage of the reaction, the intensity of the CO and CO_2_ bands both increased and developed toward an apparently stable stationary state. However, after about 10 s, the catalytic system suddenly changed drastically, before coming back to a steady state after an additional 26.7 s. At 25 °C the short-term unstable state of the Au/TiO_2_ catalytic system is characterized by a sudden increase of the CO signal, a decrease of the CO_2_ signals, and a jump of the CO vibrational band to a lower frequency (Fig. 1b–d). After that, all these values gradually return to their original values. For CO adsorption the CO vibrational band shifted to lower wavenumbers as the CO coverage increased in line with other studies of CO adsorption on Au/TiO_2_^28,29^.

The sudden increase of the CO signal after apparently reaching a stable state is intriguing. Such an increase could be due to a sudden increase in CO adsorption rate caused by a structural change of the catalyst. However, in this case, one would rather expect the CO_2_ formation rate to increase. The opposite is the case: the reaction rate actually decreases when the CO signal increases. This indicates that during this period (unstable state) the coverage of oxygen, the second reactant, not directly observable in our experiment, is decreased. This is supported by the frequency of the CO stretching vibration, which shifts to lower wavenumbers during the transient deactivation period. Lower wavenumbers of this vibrational band indicate more backdonation of electron density from the gold particles to the antibonding CO orbitals, which in turn indicates lower oxygen coverage. The duration of this unstable state is about 19 s at 25 °C. As shown in Supplementary Fig. 4, the phenomenon was also observed at a higher temperature. It should be noted that this effect was observed for many cycles when switching back and forth between the two gases.

### FTIR study of CO oxidation with different concentrations of reactants

**Fig. 2:**
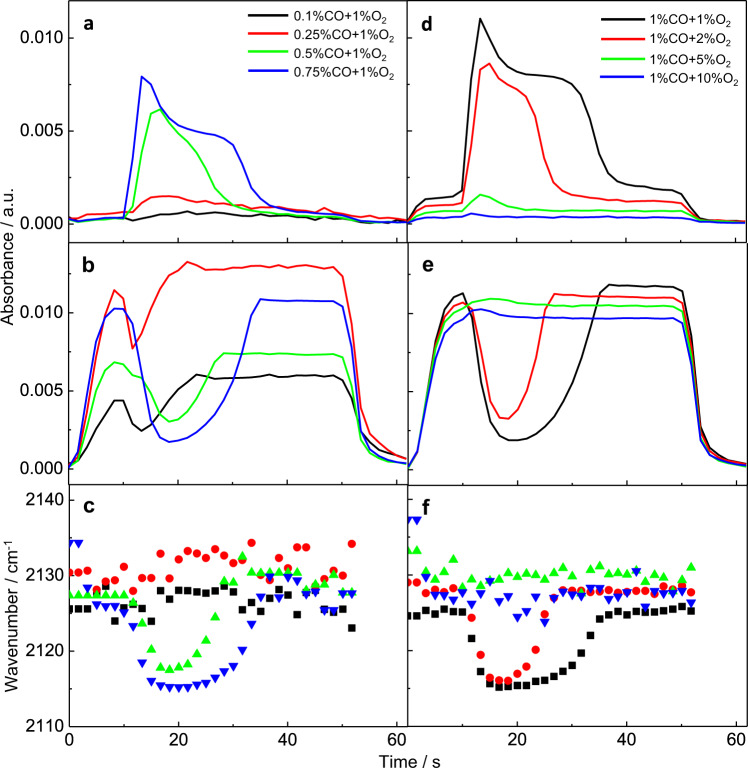
Variation of the IR intensity and wavenumber of CO-Au and CO_2_ against time during CO oxidation in different concentrations of CO and O_2_. **a**, **d** Variation of the IR intensity of CO-Au against time; **b**, **e** Variation of the IR intensity of CO_2_ against time; **c**, **f** plots of the maximum intensity of CO against time in different concentrations of CO and O_2_. The signals were corrected by subtracting the broad background signal. Experimental condition: Experimental temperature is 150 °C; flow rate is 30 mL min^−1^; modulation period is 100 s, number of spectra in one cycle is 60; during the first half-cycle CO and O_2_ in He was flowed through the cell, then the feed gas was switched to He for the second half-cycle.

To study the influence of O_2_ on the transient inactivation effect, the CO oxidation on Au/TiO_2_ was studied for various concentrations of CO (0.1–1%) and O_2_ (1–10%). The results of these experiments are shown in Fig. 2. Figure 2a–f shows the intensity of the CO and CO_2_ bands as a function of time for experiments performed at different CO and O_2_ concentrations at 150 °C. When fixing the O_2_ concentration the duration and the intensity of the effect (variation of CO and CO_2_ signals) were affected by the CO concentration. As the proportion of CO in the reactant mixture decreased, the magnitude of the effect, as measured by the variation of the CO and CO_2_ signals during the unstable state, was reduced, but it could be observed in the entire range of CO concentrations studied. When fixing the CO concentration and increasing the O_2_ concentration the system showed similar behavior in that the transient “catastrophic” event was suppressed in both duration and intensity. The results in Fig. 2a, b, d, e furthermore show that the CO_2_ and CO signals were highly inversely correlated in time. Also, the wavenumber of the CO band maximum is correlated with the CO_2_ band intensity. Note that the latter can be regarded as the reaction rate in our flow-through system.

Understanding the role of the gold NP for CO oxidation is important to develop a complete picture of the catalytic behavior of gold particles^30^. For the gold NP, many factors have been suggested to be important for the catalytic activity such as Au NP size, the charge on the Au, the contribution of low-coordinate Au active sites, and the electronic interaction of the Au with the support^4,8,14^. Both negatively charged and positively charged gold are cited to be the most active site for CO oxidation^8,13,14,30–33^. The diametrically opposite assertion complicates the understanding of the mechanism of CO oxidation on Au. Therefore, what is the charge state of Au in the unstable state and in the steady state of CO catalytic oxidation? It would be helpful to understand the transient inactivation effect in the catalytic CO oxidation over Au catalysts.

Figure 2c, f and Supplementary Fig. 6 compare the position of CO stretching band maximum in different concentrations of reactant. During the deactivation period, the CO signal increased and the CO band shifted to the red, e.g., from 2129 to 2116 cm^−1^ (Supplementary Fig. 6b). When the reaction gradually returned to the stable state, the CO band shifted back to the high wavenumber (2129 cm^−1^). For the stable state, changing the CO concentration from 0.25 to 1%, the CO-Au band of the stable state shifted from 2131 to 2124 cm^−1^ gradually (Supplementary Fig. 6a–c). While maintaining the CO concentration and increasing the O_2_ concentration from 1 to 5%, the CO-Au band shifted from 2124 back to 2129 cm^−1^ (Supplementary Fig. 6d–f). The bands with low wavenumber (e.g., 2113 cm^−1^) and high wavenumber (e.g., 2131 cm^−1^) are assigned to CO adsorption on metallic Au^0^ and CO adsorption on positively polarized gold species (Au^δ+^), respectively^34^. In addition, for the CO adsorption experiment, the CO-Au band is located at 2098 cm^−1^, indicating the gold NPs are electron rich under these conditions (Fig. 1a)^35^. Taking these results into account, we propose that the blue shift of CO band to a high wavenumber is due to the co-adsorption of O_2_ during the CO oxidation process. It has been reported that the state of Au changed continuously during the CO oxidation process. Au was positively charged under oxidizing conditions and negatively charged under reducing conditions. CO adsorbed on Au^δ+^ and reduced it to Au^0^. At the same time, CO was oxidized to CO_2_. The reduced Au^0^ could be oxidized to Au^δ+^ by adsorbed O_2_. Through rapid reduction and oxidation of the Au atoms, CO oxidation was completed^36^. Therefore, during the unstable state, the electron density or the structure of the Au particle has somehow changed, which influenced the adsorption rate of oxygen and the reaction rate on the surface. Note that the experimental conditions that change the structure of the metal NPs have been widely studied^37–44^. It was found that hydrogen adsorption could affect the electronic structure of Au NPs and lead to the rearrangement of the electronic subsystem of NPs^37,43,44^. The face-centered cubic crystal structure of Au is lost when exposing the gold sample to the hydrogen conditions^38^. Also, platinum particles could be influenced by the redox chemical environment to form twin planes^39^. Furthermore, for the Au/TiO_2_ catalyst in the CO oxidation reaction, the reconstruction of Au NPs with the major (111) and (100) facets of the gold NPs exposed by the reactants has been reported^42^. Considering the importance of the surface structure of the Au/TiO_2_ catalyst for CO oxidation, we speculate that Au/TiO_2_ may be reconstructed by the reactants at the beginning of the reaction, leading to a weakening of its ability to be oxidized by oxygen and the observed transient inactivation effects.

It should be mentioned that the accumulation of carbonate species can be excluded as the reason for the observed transient inactivation state. Supplementary Fig. 5 shows the intensity of carbonate bands at 1674, 1409, and 1247 cm^−1^ as a function of time in comparison to the band of CO_2_ at 2342 cm^−1 ^^45^. The intensity of carbonate species and CO_2_ increase or decrease synchronously, indicating that the carbonates are formed from the adsorption of CO_2_. Before the unstable state, the bands of carbonate species do not show any sudden increase that would indicate an accumulation of carbonates. For the low CO concentration experiment (Supplementary Fig. 5), the accumulation of carbonate species is very small; however, the transient unstable state is still noticeable.

### FTIR study of CO oxidation at different temperatures

**Fig. 3:**
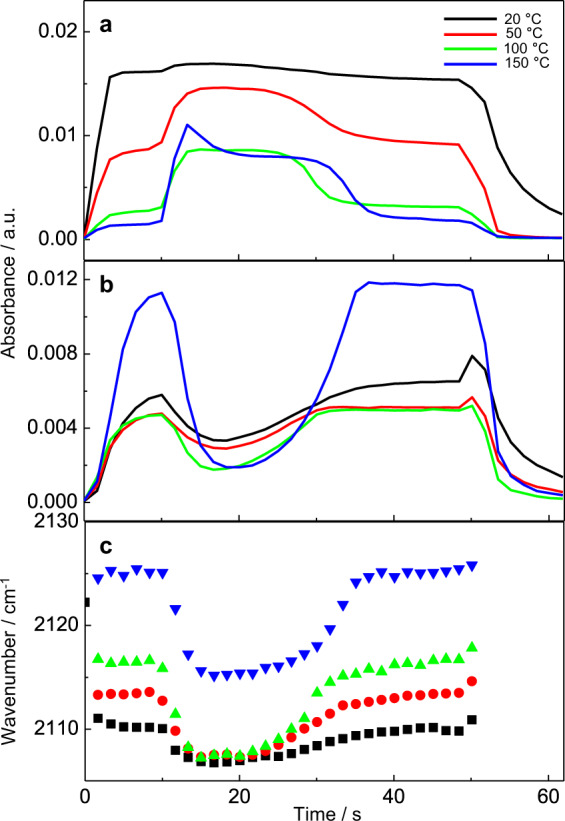
Variation of the IR intensity and wavenumber of CO-Au and CO_2_ against time during CO oxidation at different temperatures. **a** Variation of the IR intensity of CO-Au against time; **b** variation of the IR intensity of CO_2_ against time; **c** plots of the position of CO band against time at different temperatures. The signals were corrected by subtracting the broad background signal. Experimental conditions: reaction gas composition is 1% CO and 1% O_2_ balanced with He; flow rate is 30 mL min^−1^; modulation period is 100 s; number of spectra in one cycle is 60; during the first half-cycle 1% CO and 1% O_2_ in He was flowed through the cell, then the feed gas was switched to He for the second half-cycle.

Figure 3 shows the results of the CO oxidation at different temperatures (20–150 °C). Unlike concentration changes, the temperature did not affect much the duration of the transient inactivation period, but it affected the intensity of CO and CO_2_ signals, the frequency of CO stretching vibration, and the magnitude of the changes observed during this instability period. At low temperature, the CO signal in the stable steady state was higher than at high temperature, but the change in CO signal in the unstable state was much stronger at high compared to low temperature.

Based on these results, we conclude that the transient inactivation effect is affected by the concentration of reactants and temperature, especially by the ratio of CO and O_2_. Due to the fast reaction of CO and O_2_ on the catalyst surface in the first stabilization stage, the rapid consumption of adsorbed O_2_ and the slow supply of new oxygen causes a transient “catastrophic” event on the Au/TiO_2_ surface. In an unstable state, the structure of Au catalyst may change.

### UV-vis study of CO adsorption and CO oxidation

**Fig. 4:**
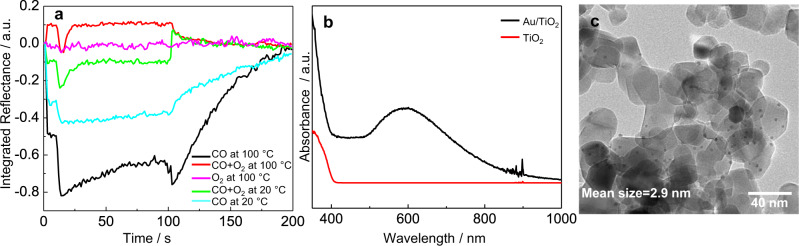
In situ UV-vis signal as a function of time, diffuse reflectance UV-vis spectra of TiO_2_ and Au/TiO_2_, and TEM image of Au/TiO_2_. **a** UV-vis signal (integrated reflectance between 750 and 950 nm) as a function of time during CO oxidation, CO adsorption, and O_2_ adsorption at 20 and 100 °C. Experimental condition: 30 mL min^−1^ of 1% CO and/or 1% O_2_ in He were flowed through the cell during the first half-cycle; the modulation period was 200 s; 8 cycles were measured and averaged to enhance the signal. **b** Diffuse reflectance UV-vis spectra of TiO_2_ and Au/TiO_2._ **c** TEM image of Au/TiO_2_.

To shed light on the influence of the reactants on the structure of the catalyst, UV-vis reflectance spectroscopy of the catalyst under reaction conditions was applied. UV-vis spectroscopy is a powerful method for electronic structure studies as it can probe, for example, the electronic d–d transitions for transition metals present in the catalysts^13^. The optical properties of the Au NPs are significantly affected by their structure, e.g., size, shape, etc.^46–49^. For example, both size and concentration of gold NPs could be directly determined by UV-vis spectroscopy^46^. The surface plasmon resonance (SPR) was modified and shifted to a high wavelength when the particles deviated from spherical geometry^49,50^. These examples demonstrate that UV-vis spectroscopy is a powerful tool for monitoring the change in Au NP structure. The comparison between the UV-vis spectra of the catalyst and TiO_2_ support (Fig. 4b) shows that the Au particles lead to increased absorption extending till about 1000 nm with a broad peak at about 600 nm. The latter can be attributed to the SPR of the gold particles. For the Au/TiO_2_, a broad absorption centered at 600 nm is typically assigned to the PR in the Au NPs^51,52^. The peak is not very pronounced in our case in agreement with the small size of the particles, see transmission electron microscopy (TEM) image in Fig. 4c and Supplementary Fig. 7. The particles with a mean diameter of 2.9 nm are well separated. XPS spectra of Au 4*f* and Ti 2*p* indicate metallic Au^0^ and Ti^4+^ (Supplementary Fig. 8)^53^. X-ray diffraction (XRD) spectra show a diffraction peak at 44.0°, which can be indexed as gold (200) reflection (Supplementary Fig. 9)^54^. Note that we did not find any indication of strong metal-support interaction in our system (Supplementary Figs. 11–13).

When exposing the Au/TiO_2_ catalyst to the reactant (CO alone or a CO + O_2_ mixture), the absorption spectrum showed a change in a broad range between 600 and 1000 nm. This broad signal has been assigned to the SPR of the gold NPs^55^, indicating the electronic properties of the Au have changed.

To explore the influence of reactants on the Au particle structure, the integrated reflectance between 750 and 950 nm was used to follow the spectral changes as a function of time during the modulation experiment (Fig. 4a). The reason that we use the reflectance signal between 750 and 950 nm is that the UV-vis spectra change in intensity obviously in this frequency range but did not change much in the range between 500 and 600 nm (Supplementary Fig. 10). Considering that the peak centered at 600 nm is very broad, we believe that this peak consists of multiple small peaks. The reflectance at low wavelength did not change with the reactants, indicating that only part of the NPs was affected rather than all. The integrated reflectance between 750 and 950 nm is similar to the region between 500 and 950 nm (Supplementary Fig. 10). Note that these experiments were performed in the same small-volume cell as the DRIFT experiments. The change of gas from He to CO and O_2_ at 100 °C had a small but significant effect on the optical properties of the catalyst (red line in Fig. 4a). The increase in reflectance was reversible and disappeared once the cell was loaded with He again. For the flow of CO + O_2_, after about 10 s, a sharply declining and then rising signal was detected, which was consistent with the transient inactivation effect in the IR spectrum. This phenomenon suggests that the structure of the Au particles was changed during the unstable state. Interestingly, the optical properties already change immediately when introducing the CO/O_2_ mixture. They change again in the deactivation period. When changing the gas back to He the optical properties of the catalyst changed again, returning to the initial values. This return to the initial value has a fast (seconds) but also a slow (tens of seconds) component.

To clarify which reactant causes the change in optical properties (the reconstruction of Au particles), CO and O_2_ were separately introduced to the catalyst surface. The O_2_ had no influence on the change of UV-vis spectrum but the CO caused the Au signal to decrease. About 10 s after changing the flow to CO, the appearance of a rapid drop in intensity for the Au signal indicates that the transient inactivation effect is due to the reconstruction of the catalyst by CO. At room temperature, the Au signal of both CO adsorption and CO oxidation showed a similar trend to the CO adsorption at 100 °C. The different intensity of Au signals indicates structural change is more pronounced at high temperature. The different absorption spectra of CO + O_2_ and CO at high temperatures suggest that O_2_ could also affect the Au structure when CO is present in the reactant. CO may also play the role of activating gold sites. The observation that oxygen alone had no influence on the optical properties of the catalyst indicates that the changes are not only due to modifications of the electronic properties of the metal particles but also supports the hypothesis that the changes are caused by structural changes induced by adsorbed CO.

To confirm the role of O_2_ in the Au/TiO_2_ reconstruction, the WGS reaction was studied in the same cell. As shown in Supplementary Fig. 14, the Au/TiO_2_ was very active in the WGS reaction. The conversion of CO is over 90% at 260 °C. But the unstable state is absent in the spectra of CO + H_2_O reaction at various temperatures (Supplementary Fig. 15). These results suggest that O_2_ is crucial for the reconstruction of the Au NPs.

Taking the results of the IR and UV-vis spectra into account, we propose that (i) the reconstruction of the Au particles leads to a transient inactivation effect of CO oxidation on the Au/TiO_2_ surface. (ii) The reconstruction is mainly caused by CO, but does not affect the pure CO adsorption on Au (seen in Fig. 1). (iii) In the presence of CO O_2_ also participates in the reconstruction of Au.

In conclusion, we studied CO oxidation on Au/TiO_2_ on a short time scale after admitting the reactants. The spectroscopic results indicate a transient inactivation effect due to the Au reconstruction by the reactants at the beginning of the reaction. The transient inactivation effect reflects the state change of Au particles during CO oxidation. The effect is fully reversible, i.e. it is observed in subsequent cycles of admitting and removing the reactant mixture. More importantly, due to the structural change, the adsorption of oxygen on Au/TiO_2_ is hindered, meanwhile leading to lower CO oxidation activity before returning to a steady state characterized by better catalytic activity.

## Methods

### Catalyst preparation

The Au/TiO_2_ catalyst (1% Au) was prepared by a deposition-precipitation method^56,57^. Typically, a certain amount of HAuCl_4_ was dissolved in 100 mL of water. Then the pH of HAuCl_4_ solution was adjusted to 8 by adding NaOH solution (1 mol L^−1^). After that, 1 g TiO_2_ (P25) support was added under vigorous stirring. The mixture was heated to 70 °C and kept for 3 h during which the pH value was maintained at 8. Finally, the resulting precipitate was filtered, washed with deionized water, then dried at 80 °C overnight and calcined at 300 °C for 1 h.

### Characterizations

DRIFT spectra were measured on a Bruker Equinox 55 FTIR spectrometer equipped with a liquid-nitrogen-cooled MCT detector at 8 cm^–1^ resolution. Catalyst powder was placed in a home-built cell (volume = 67.4 μL). The inlet of the cell was connected to an automated 4-way valve (VICI Valco). Before catalytic reaction, the sample was pretreated in situ by flowing He at 80 °C for 1 h. MES experiments were carried out by periodically switching between two different gas atmospheres according to the following sequences: (i) for CO adsorption, 1% of CO in He and pure He; and (ii) for CO oxidation, 1% of CO and 1% of O_2_ in He and He. The flow rate was 30 mL min^−1^. For each MES measurement, two dummy cycles (without data acquisition) were executed before averaging six cycles to enhance the S/N ratio^58,59^. The modulation period is 100 s and the number of spectra in one cycle is 60. Thirty-two scans were averaged per spectrum in each cycle. The flowing rate is 30 mL min^−1^. For the CO oxidation, during the first half-cycle CO and O_2_ in He was flowed through the cell, then the feed gas was switched to He for the second half-cycle. For the CO adsorption, during the first half-cycle 1% CO in He was flowed through the cell, then the feed gas was switched to He for the second half-cycle. It should be noted that we did not perform a PSD in this study. The phase domain data do not easily provide information about the rapidly changing signals observed here during the unstable state. For the WGS reaction, during the first half-cycle CO and H_2_O in He was flowed through the cell, then the feed gas was switched to He for the second half-cycle.

UV-vis spectroscopy (AVASPEC-ULS2048CL-EVO-RS with a Balanced Deuterium-Halogen light source) was used to in situ record the structural change of Au/TiO_2_. A fiber optic reflection probe bundle (Thorlabs RP28) was used for reflectivity measurements and was placed close to the sample. The light from the source is guided to the catalyst, and the reflected light is collected and sent through the fiber to the spectrometer. The gas flow is the same as in the DRIFT experiments described above. The UV-vis reflectance data were obtained by taking the mean of the spectra in the wavelength range from 750 to 950 nm (other wavelength ranges did not show temporal changes other than baseline drifts). A temporal baseline drift in the spectra was corrected by fitting and subtracting a polynomial (2nd order) to this time-dependence. In continuation, the mean of the multiple repetitions of the experiment (4 or 8) was taken and the first value was subtracted, in order to show changes with respect to the state before switching.

Diffuse reflectance UV-vis of samples was obtained using a Jasco V-670 spectrometer. TEM pictures were obtained on TEM-Tecnai G2 and Philips CM30 with an accelerating voltage of 150 kV. Powder XRD was carried out on a Philips diffractometer of X’pert Company with mono-chromatized Cu Kα radiation (λ = 1.5406 Å). XPS measurements were performed using a Physical Electronics Versa Probe III system with a hemispherical analyzer and monochromatic Al Kα source. The energy scale linearity was calibrated with Au 4*f*_7/2_ at 83.97 eV and Cu 2*p*_3/2_ 932.60 eV (±0.1 eV). All data were measured at room temperature with a pass energy of 55 eV, at a take-off angle of 45°, and an angular acceptance angle of ±20°. The samples were pressed into a double-sided tape and were electrically isolated during measurement. A low-energy dual beam charge compensation system, using an argon ion gun and electron flood gun, was used to mitigate the effects of charging. The X-ray beam size on the sample was ~100 µm.

## Supplementary information

Supplementary Information

## Data Availability

The data that support the findings of this study are available within the paper and its Supplementary information, and all data are available from the authors upon request. The spectroscopic data generated in this study have been deposited in the Zenodo repository under accession code (Zenodo) (10.5281/zenodo.7024765).

## References

[1] Tang H (2017). Classical strong metal–support interactions between gold nanoparticles and titanium dioxide. Sci. Adv..

[2] Chen BWJ, Xu L, Mavrikakis M (2021). Computational methods in heterogeneous catalysis. Chem. Rev..

[3] Védrine, J. C. Heterogeneous catalysis on metal oxides. *Catalysts* **7**, 341 (2017).

[4] Liu L, Corma A (2018). Metal catalysts for heterogeneous catalysis: from single atoms to nanoclusters and nanoparticles. Chem. Rev..

[5] Haruta M (1997). Size- and support-dependency in the catalysis of gold. Catal. Today.

[6] Nkosi B, Adams MD, Coville NJ, Hutchings GJ (1991). Hydrochlorination of acetylene using carbon-supported gold catalysts: a study of catalyst reactivation. J. Catal..

[7] Hutchings GJ (1985). Vapor phase hydrochlorination of acetylene: correlation of catalytic activity of supported metal chloride catalysts. J. Catal..

[8] Sankar M (2020). Role of the support in gold-containing nanoparticles as heterogeneous catalysts. Chem. Rev..

[9] Herzing AA, Kiely CJ, Carley AF, Landon P, Hutchings GJ (2008). Identification of active gold nanoclusters on iron oxide supports for CO oxidation. Science.

[10] Valden M, Lai X, Goodman DW (1998). Onset of catalytic activity of gold clusters on titania with the appearance of nonmetallic properties. Science.

[11] Green IX, Tang W, Neurock M, Yates JT (2011). Spectroscopic observation of dual catalytic sites during oxidation of CO on a Au/TiO_2_ catalyst. Science.

[12] Saavedra J, Pursell CJ, Chandler BD (2018). CO oxidation kinetics over Au/TiO_2_ and Au/Al_2_O_3_ catalysts: evidence for a common water-assisted mechanism. J. Am. Chem. Soc..

[13] Zhou X (2018). Unraveling charge state of supported Au single-atoms during CO oxidation. J. Am. Chem. Soc..

[14] Ishida T, Murayama T, Taketoshi A, Haruta M (2020). Importance of size and contact structure of gold nanoparticles for the genesis of unique catalytic processes. Chem. Rev..

[15] Martínez-Huerta MV, Deo G, Fierro JLG, Bañares MA (2008). Operando Raman-GC study on the structure−activity relationships in V^5+^/CeO_2_ catalyst for ethane oxidative dehydrogenation: the formation of CeVO_4_. J. Phys. Chem. C..

[16] Tinnemans SJ (2006). Combining operando techniques in one spectroscopic-reaction cell: new opportunities for elucidating the active site and related reaction mechanism in catalysis. Catal. Today.

[17] Brückner A, Kondratenko E (2006). Simultaneous operando EPR/UV–vis/laser–Raman spectroscopy—a powerful tool for monitoring transition metal oxide catalysts during reaction. Catal. Today.

[18] Meunier FC, Goguet A, Shekhtman S, Rooney D, Daly H (2008). A modified commercial DRIFTS cell for kinetically relevant operando studies of heterogeneous catalytic reactions. Appl. Catal..

[19] Thibault-Starzyk F (2009). Real-time infrared detection of cyanide flip on silver-alumina NO_x_ removal catalyst. Science.

[20] Wille A, Fridell E (2007). Millisecond step-scan FT-IR transmission spectroscopy under transient reaction conditions: CO oxidation over Pt/Al_2_O_3_. Appl. Catal. B.

[21] Guzman F, Chuang SSC (2010). Tracing the reaction steps involving oxygen and IR observable species in ethanol photocatalytic oxidation on TiO_2_. J. Am. Chem. Soc..

[22] Zhou Z-Y, Sun S-G (2005). In situ step-scan time-resolved microscope FTIR spectroscopy applied in irreversible electrochemical reactions. Electrochim. Acta.

[23] Dal Santo V (2005). Fast transient infrared studies in material science: development of a novel low dead-volume, high temperature DRIFTS cell. Talanta.

[24] Maeda N, Hungerbühler K, Baiker A (2011). Asymmetric hydrogenation on chirally modified Pt: origin of hydrogen in the N–H–O interaction between cinchonidine and ketone. J. Am. Chem. Soc..

[25] Powell CD, Daigh AW, Pollock MN, Chandler BD, Pursell CJ (2017). CO adsorption on Au/TiO_2_ catalysts: observations, quantification, and explanation of a broad-band infrared signal. J. Phys. Chem. C..

[26] Li H (2022). Switching the nonlinear optical absorption of titanium carbide MXene by modulation of the surface terminations. ACS Nano.

[27] Debeila MA, Coville NJ, Scurrell MS, Hearne GR (2002). DRIFTS studies of the interaction of nitric oxide and carbon monoxide on Au–TiO_2_. Catal. Today.

[28] Boccuzzi F, Chiorino A, Tsubota S, Haruta M (1996). FTIR study of carbon monoxide oxidation and scrambling at room temperature over gold supported on ZnO and TiO_2_. 2. J. Phys. Chem..

[29] Grunwaldt J-D, Maciejewski M, Becker OS, Fabrizioli P, Baiker A (1999). Comparative study of Au/TiO_2_ and Au/ZrO_2_ catalysts for low-temperature CO oxidation. J. Catal..

[30] Bond, G. C., Louis, C., & Thompson D. T. *Catalysis by Gold* (Imperial College Press and World Scientific Publishing Co., 2006).

[31] Yoshida T (2018). Carbon monoxide oxidation by polyoxometalate-supported gold nanoparticulate catalysts: activity, stability, and temperature-dependent activation properties. Angew. Chem. Int. Ed..

[32] Quinet E (2009). On the mechanism of hydrogen-promoted gold-catalyzed CO oxidation. J. Catal..

[33] Min BK, Friend CM (2007). Heterogeneous gold-based catalysis for green chemistry: low-temperature CO oxidation and propene oxidation. Chem. Rev..

[34] Green IX, Tang W, McEntee M, Neurock M, Yates JT (2012). Inhibition at perimeter sites of Au/TiO_2_ oxidation catalyst by reactant oxygen. J. Am. Chem. Soc..

[35] Chen M, Cai Y, Yan Z, Goodman DW (2006). On the origin of the unique properties of supported Au nanoparticles. J. Am. Chem. Soc..

[36] Wang Y-G, Yoon Y, Glezakou V-A, Li J, Rousseau R (2013). The role of reducible oxide–metal cluster charge transfer in catalytic processes: new insights on the catalytic mechanism of CO oxidation on Au/TiO_2_ from ab initio molecular dynamics. J. Am. Chem. Soc..

[37] Gatin AK, Grishin MV, Dokhlikova NV, Kolchenko NN, Shub BR (2016). The effect of hydrogen adsorption on the electronic structure of gold nanoparticles. Doc. Phys. chem..

[38] Nassereddine A (2021). Revealing size dependent structural transitions in supported gold nanoparticles in hydrogen at atmospheric pressure. Small.

[39] Frey H, Beck A, Huang X, van Bokhoven JA, Willinger MG (2022). Dynamic interplay between metal nanoparticles and oxide support under redox conditions. Science.

[40] Hansen Thomas W (2001). Atomic-resolution in situ transmission electron microscopy of a promoter of a heterogeneous catalyst. Science.

[41] Kuwauchi Y (2013). Stepwise displacement of catalytically active gold nanoparticles on cerium oxide. Nano Lett..

[42] Kuwauchi Y, Yoshida H, Akita T, Haruta M, Takeda S (2012). Intrinsic catalytic structure of gold nanoparticles supported on TiO_2_. Angew. Chem. Int. Ed..

[43] Silverwood IP, Rogers SM, Callear SK, Parker SF, Catlow CRA (2016). Evidence for a surface gold hydride on a nanostructured gold catalyst. Chem. Commun..

[44] Wu B (2016). Anisotropic growth of TiO_2_ onto gold nanorods for plasmon-enhanced hydrogen production from water reduction. J. Am. Chem. Soc..

[45] Wang Y (2017). Avoiding self-poisoning: a key feature for the high activity of Au/Mg(OH)_2_ catalysts in continuous low-temperature CO oxidation. Angew. Chem. Int. Ed..

[46] Haiss W, Thanh NTK, Aveyard J, Fernig DG (2007). Determination of size and concentration of gold nanoparticles from UV−vis spectra. Anal. Chem..

[47] Jensen T, Kelly L, Lazarides A, Schatz GC (1999). Electrodynamics of noble metal nanoparticles and nanoparticle clusters. J. Clust. Sci..

[48] Kelly KL, Coronado E, Zhao LL, Schatz GC (2003). The optical properties of metal nanoparticles: the influence of size, shape, and dielectric environment. J. Phys. Chem. B.

[49] Ghosh SK, Pal T (2007). Interparticle coupling effect on the surface plasmon resonance of gold nanoparticles: from theory to applications. Chem. Rev..

[50] Orendorff CJ, Sau TK, Murphy CJ (2006). Shape-dependent plasmon-resonant gold nanoparticles. Small.

[51] Borensztein Y, Delannoy L, Djedidi A, Barrera RG, Louis C (2010). Monitoring of the plasmon resonance of gold nanoparticles in Au/TiO_2_ catalyst under oxidative and reducing atmospheres. J. Phys. Chem. C..

[52] Gonzalez-Yañez EO, Fuentes GA, Hernández-Terán ME, Fierro-Gonzalez JC (2013). Influence of supported gold particles on the surface reactions of ethanol on TiO_2_. Appl. Catal..

[53] Wang X, Maeda N, Baiker A (2016). Synergistic effects of Au and FeO_x_ nanocomposites in catalytic NO reduction with CO. ACS Catal..

[54] Youssef AM, Abdel-Aziz MS, El-Sayed SM (2014). Chitosan nanocomposite films based on Ag-NP and Au-NP biosynthesis by *Bacillus subtilis* as packaging materials. Int. J. Biol. Macromol..

[55] Schilling C, Hess C (2018). Real-time observation of the defect dynamics in working Au/CeO_2_ catalysts by combined operando Raman/UV–vis spectroscopy. J. Phys. Chem. C..

[56] Zhang Y (2020). Boosting the catalysis of gold by O_2_ activation at Au-SiO_2_ interface. Nat. Commun..

[57] Tang H (2016). Ultrastable hydroxyapatite/titanium-dioxide-supported gold nanocatalyst with strong metal–support interaction for carbon monoxide oxidation. Angew. Chem. Int. Ed..

[58] Maeda N, Meemken F, Hungerbühler K, Baiker A (2013). Selectivity-controlling factors in catalytic methanol amination studied by isotopically modulated excitation IR spectroscopy. ACS Catal..

[59] Wang, X., Maeda, N., Meier, D. M. & Baiker, A. Bimetallic AuPd@CeO_2_ nanoparticles supported on potassium titanate nanobelts: a highly efficient catalyst for the reduction of NO with CO. *Catal. Lett*. **151**, 2483–2491 (2021).

