# Supplementary material for: Insight into the transient inactivation effect on Au/TiO2 catalyst by in-situ DRIFT and UV–vis spectroscopy
Source: Nat Commun. 2022 Sep 17;13:5458. doi: 10.1038/s41467-022-33187-y (PMC9482617; doi:10.1038/s41467-022-33187-y)
Supplement: Supplementary file 1 — Supplementary Information [file 41467_2022_33187_MOESM1_ESM.docx]

**Insight into the transient inactivation effect on Au/TiO_2_ catalyst by in-situ DRIFT and UV–vis spectroscopy**

Xianwei Wang*, Arnulf Rosspeintner, Abolfazl Ziarati, Jiangtao Zhao, Thomas Bürgi*

 Department of Physical Chemistry, University of Geneva, 1211 Geneva 4, Switzerland

Email: Xianwei Wang ([Xianwei.wang@unige.ch](mailto:Xianwei.wang@unige.ch)); Thomas Bürgi ([Thomas.Buergi@unige.ch](mailto:Thomas.Buergi@unige.ch))

**FTIR spectra of CO adsorption**


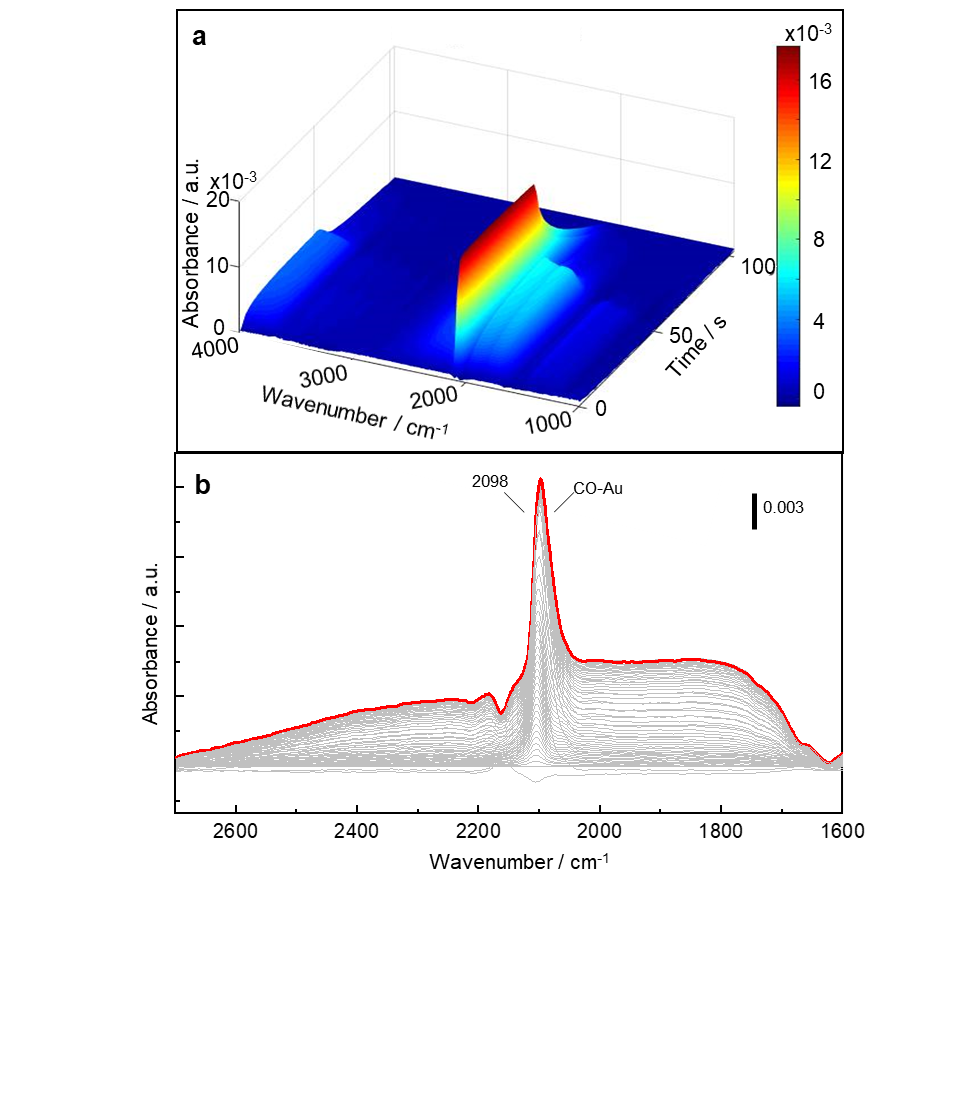


**Supplementary Figure 1.** **3D and** **normal FTIR spectra for CO adsorption on Au/TiO_2_ at 25 °C.** (**a**) 3D view; (**b**) normal spectra. Experimental conditions: Flow rate was 30 ml min^-1^; the modulation period was 100 s; The number of spectra in one cycle was 60; For the first 30 spectra, 1% CO in He was flowed through the cell, and then the feed gas was switched to He for the second half-cycle.


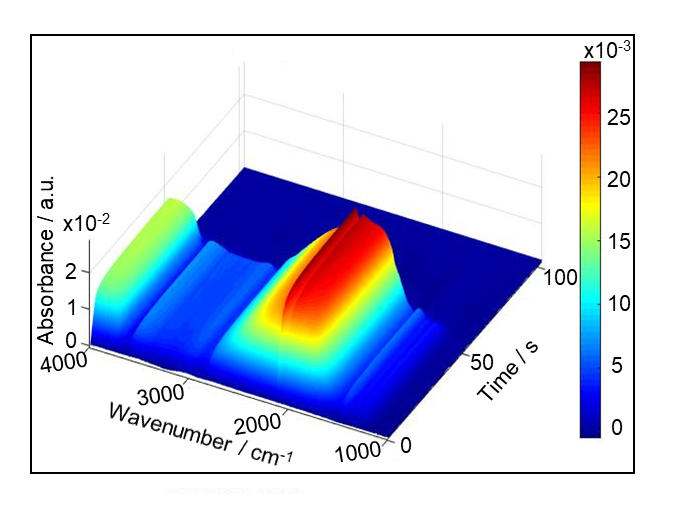


**Supplementary Figure 2.** **3D FTIR spectra of CO adsorption on Au/TiO_2_ at 150 °C.** Experimental conditions: Flow rate was 30 ml min^-1^; the modulation period was 100 s; the number of spectra in one cycle was 60; for the first 30 spectra, 1% CO in He was flowed through the cell, and then the feed gas was switched to He for the second half-cycle.

**Evolution of CO_2_ band in the cell**


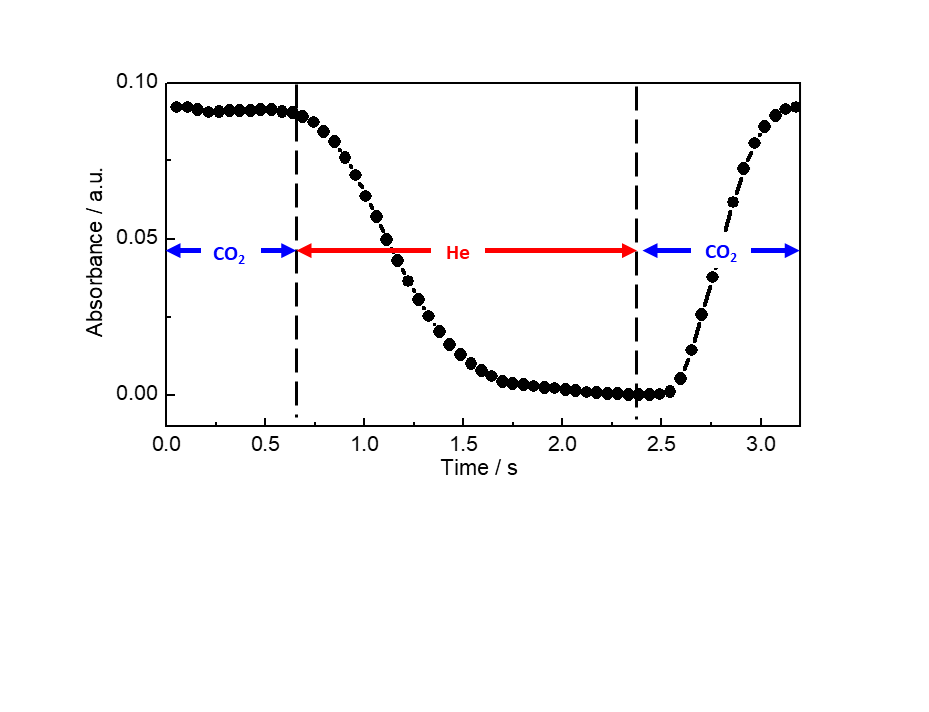


**Supplementary Figure 3.** **Plot of CO_2_ signal at 2342 cm^-1^ as function of time.** Experimental conditions: Flow rate was 30 ml min^-1^; modulation period was 3.2s; number of spectra in one cycle was 60; during the first half-cycle pure CO_2_ was flowed through the cell, then the feed gas was switched to He for the second half-cycle; Sodium Bromide (KBr) powder was used as the substrate for diffuse reflection. Note that there is a time lag of about 2.4 s between the switching of the valve and the arrival of the gas in the DRIFT cell.

**FTIR Spectra of CO oxidation**


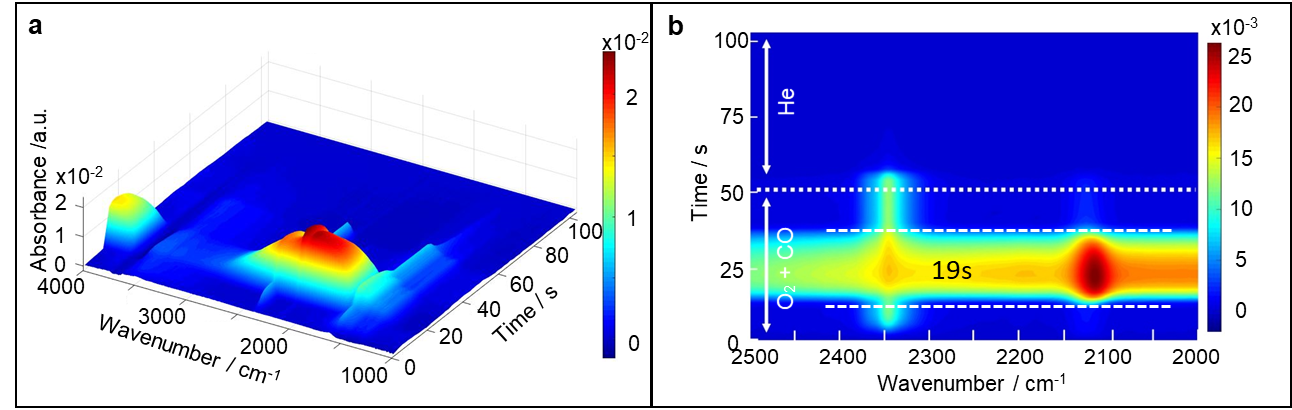


**Supplementary Figure 4.** **3D and top view of FTIR spectra for CO oxidation over Au/TiO_2_ at 150 °C.** (**a**) 3D view; (**b**) top view. Experimental conditions: Flow rate was 30 ml min^-1^; modulation period was 100 s; number of spectra in one cycle was 60; during the first half-cycle 1% CO and 1% O_2_ in He was flowed through the cell, then the feed gas was switched to He for the second half-cycle.

**FTIR spectra of carbonate species and CO_2_**


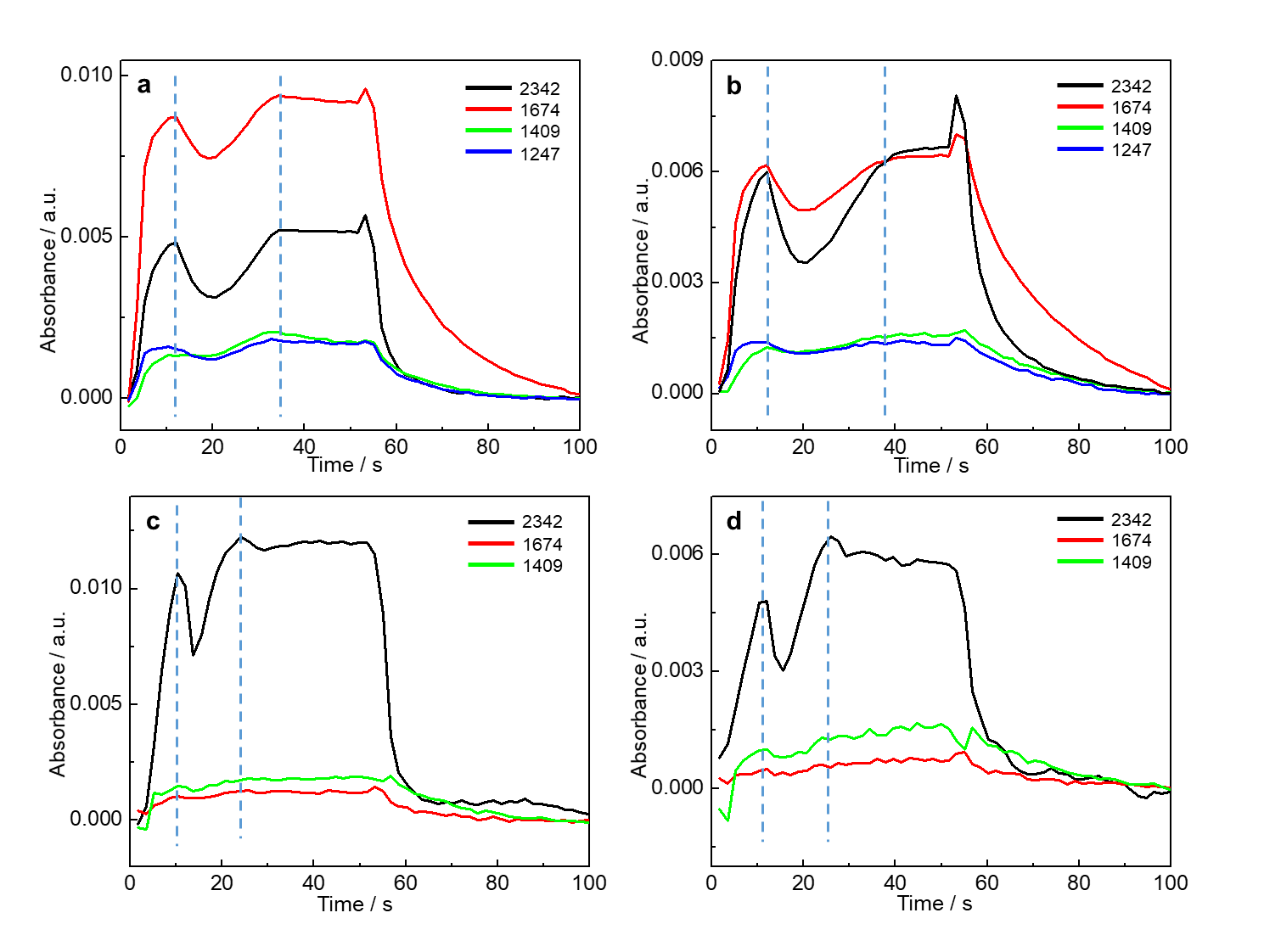


**Supplementary Figure 5. Plots of carbonate species and CO_2_ band intensities against time for different reaction conditions.** (**a**) 50 °C and 1% of CO and 1% of O_2_, (**b**) 20 °C and 1% of CO and 1% of O_2_, (**c**) 150 °C, 0.25% of CO and 1% of O_2_ and (**d**) 150 °C, 0.1% of CO and 1% of O_2_. Other experimental conditions: Flow rate was 30 ml min^-1^; modulation period was 100 s; number of spectra in one cycle was 60; during the first half-cycle CO and O_2_ in He was flowed through the cell, then the feed gas was switched to He for the second half-cycle. Numbers in the legend refer to the wavenumber of the corresponding band.


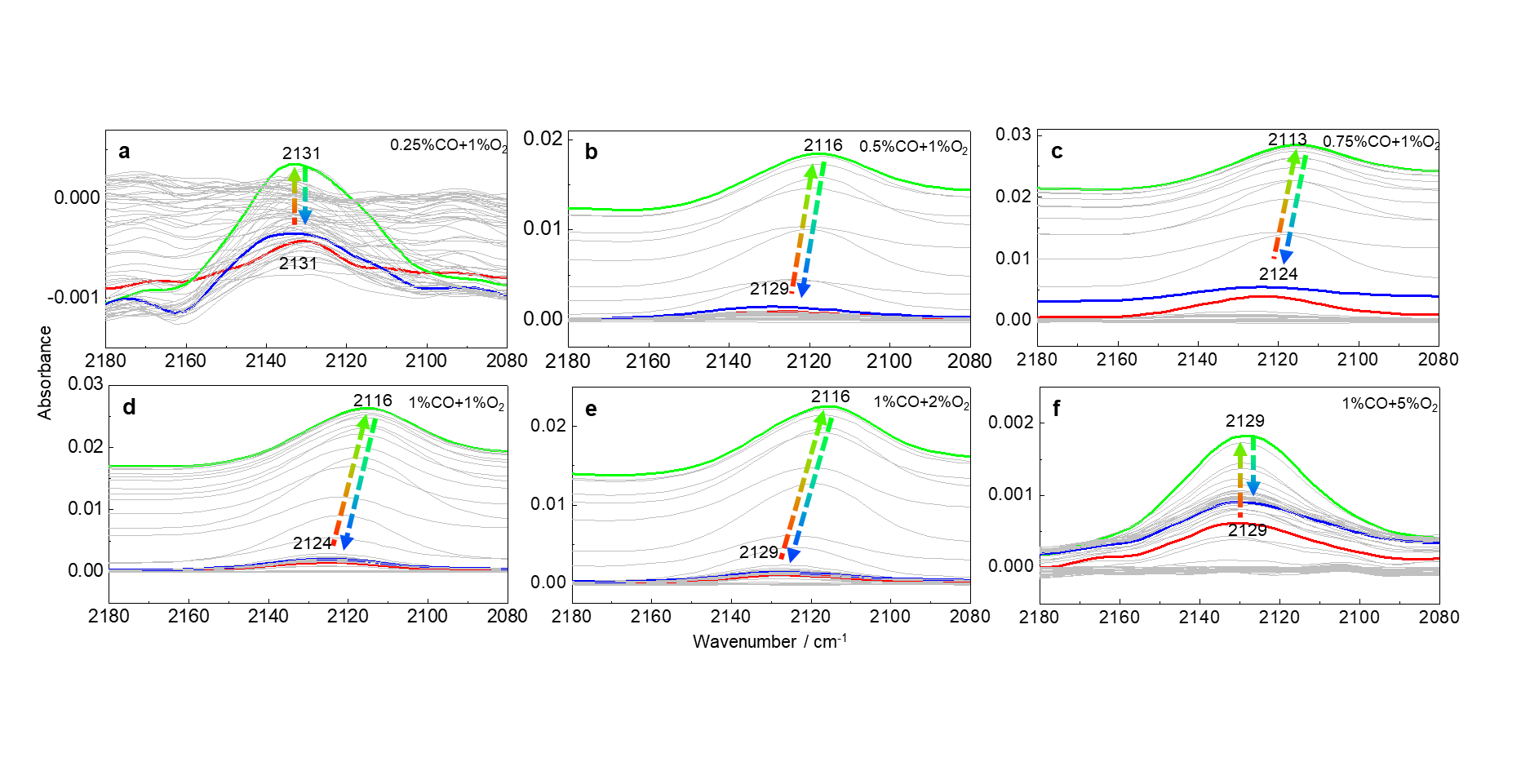


**Supplementary Figure 6.** **IR spectra of CO-Au recorded during the CO oxidation process at different concentrations of CO and O_2_.** (**a**) 0.25% of CO and 1% of O_2_; (**b**) 0.5% of CO and 1% of O_2_; (**c**) 0.75% of CO and 1% of O_2_; (**d**) 1% of CO and 1% of O_2_; (**e**) 1% of CO and 2% of O_2_; (**f**)1% of CO and 5% of O_2_. The red spectra are the signals before the unstable state. The green spectra are the maximum signal of the unstable state. The blue spectra are the signal after unstable state. Experimental conditions: Experimental temperature is 150 °C; flowing rate was 30 ml min^-1^; modulation period was 100 s; number of spectra in one cycle was 60; during the first half-cycle CO and O_2_ in He was flowed through the cell, then the feed gas was switched to He for the second half-cycle.

**TEM data of Au/TiO_2_**


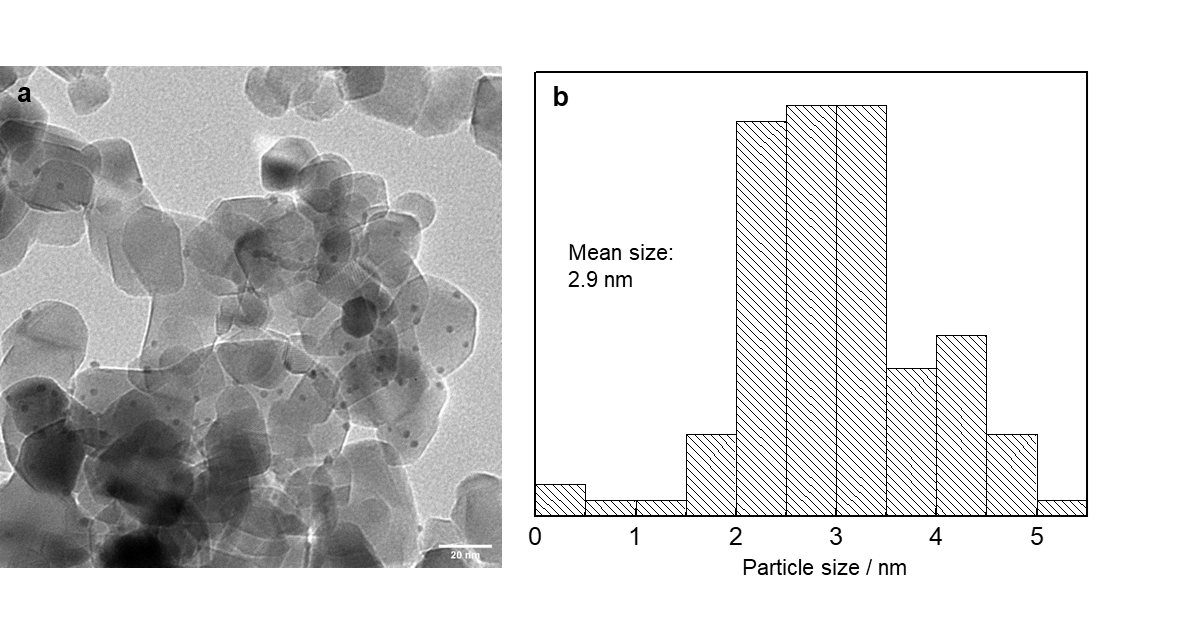


**Supplementary Figure 7.** **TEM image and distribution of Au particle size of Au/TiO_2_ sample.** (**a**) TEM image; (**b**) distribution of Au particle size.

**XPS data of Au/TiO_2_**


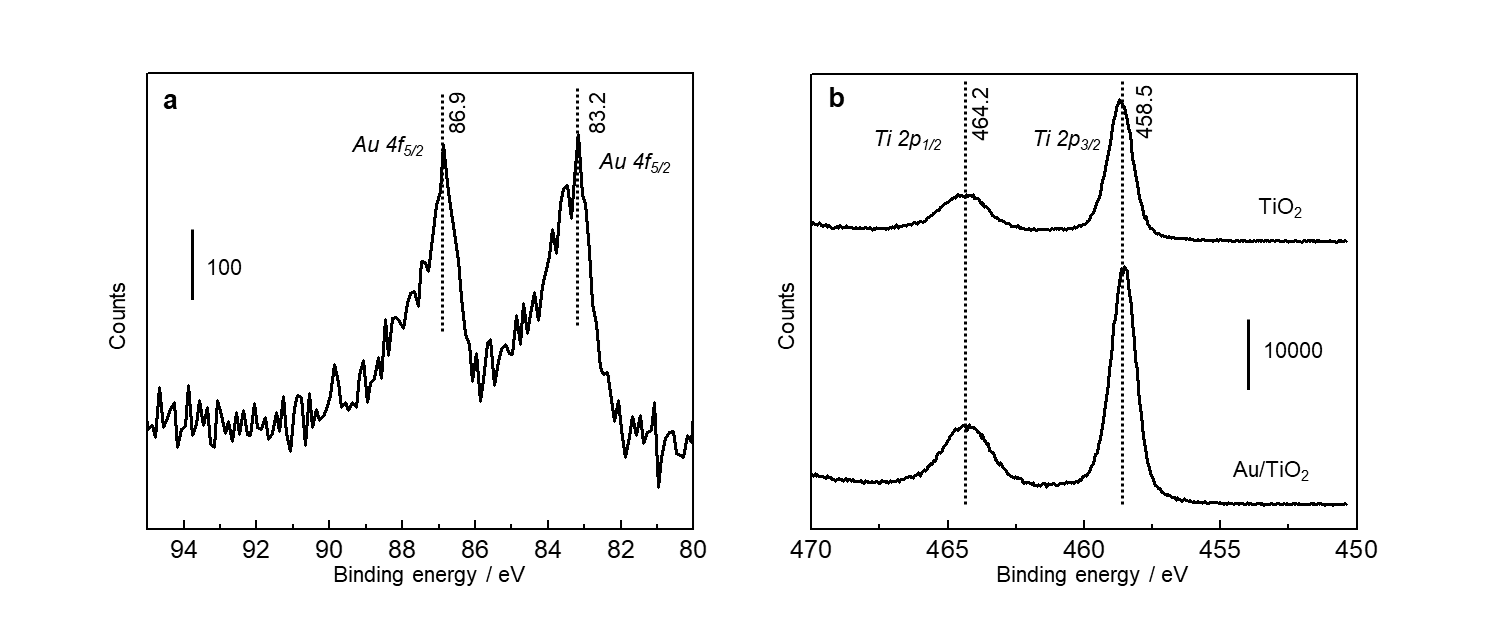


**Supplementary Figure 8.** **XPS spectra of fresh Au/TiO_2_.** (**a**) XPS spectra of Au 4*f*; (**b**) XPS spectra of Ti 2*p*.

**XRD data of Au/TiO_2_**


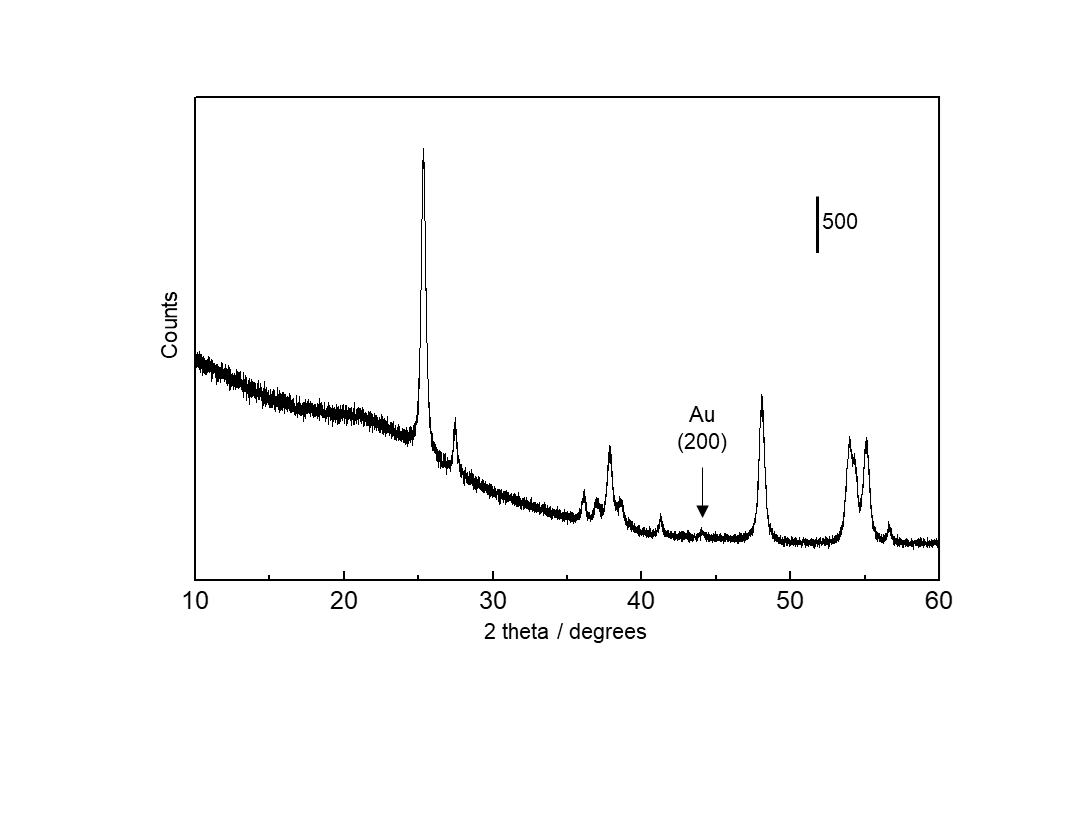


**Supplementary Figure 9.** **XRD spectrum of fresh Au/TiO_2_.** The diffraction peak at 44° is assigned to gold (200) reflection.

**Evolution of in-situ UV-vis signal in different wavelength regions**


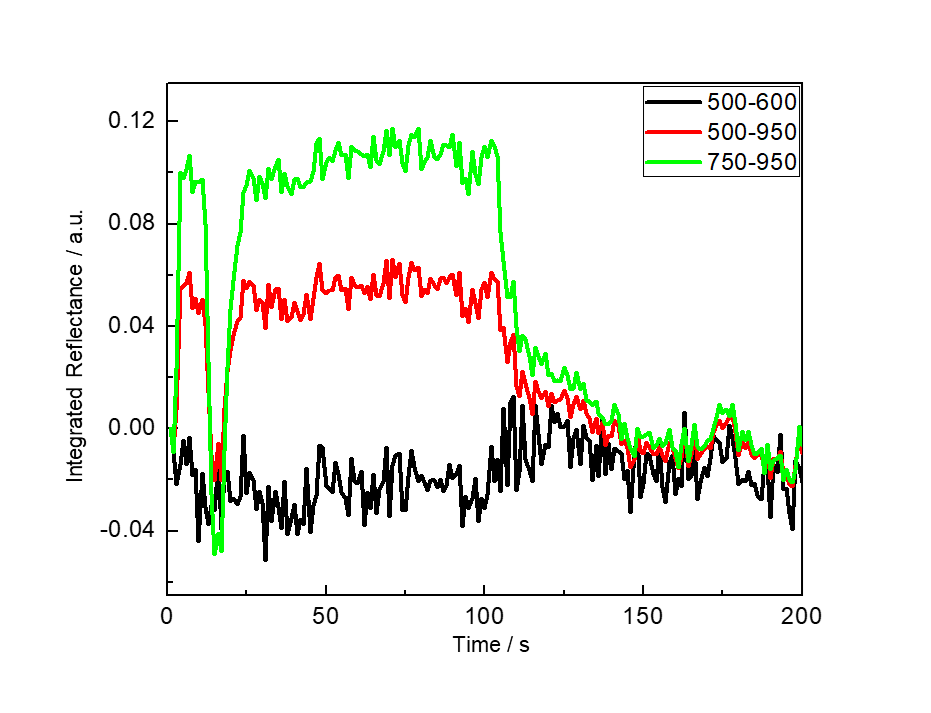


**Supplementary Figure 10.** **Integrated reflectance of UV-vis signal at different wavelength intervals as a function of time for CO oxidation at 100 °C.** Experimental condition: 100 °C; gas flow: 1% CO and 1% O2 in He were flowed through the cell during the first half-cycle, then the gas flow was changed to He; the modulation period was 200 s; 30 cycles were measured and averaged to enhance the signal; flow rate was 30 ml/min.

**Study of strong metal support interaction over Au/TiO_2_**

Before the FTIR measurement, the fresh Au/TiO_2_ was pretreated under different conditions to study the SMSI effect. The fresh sample was reduced under 10% H_2_/He at 500 °C and denoted as Au/TiO_2_-H500. Some Au/TiO_2_-H500 samples were further pretreated under 10% O_2_/He at 400°C and donated as Au/TiO_2_-H500-O400.

Supplementary Figure 11 shows the DRIFT spectra of CO adsorption on various Au/TiO_2_ samples with different pretreatments. In Supplementary Figure 11a band at 2101 cm^-1^ assigned to CO adsorption on metallic Au (CO-Au^0^) emerged for the fresh Au/TiO_2_^1^. For the classical SMSI, after pretreatment with H_2_ at 500 °C, CO adsorption will be suppressed and disappear from the spectrum due to encapsulation by the titania overlayer^2^. But in Supplementary Figure 11b, the band at 2101 cm^-1^ could still be observed at the first stage of CO adsorption. After some time a band at 2073 cm^-1^ ascribed to the negatively charged Au (CO-Au*^x^*^−^) emerged^2^. In the meanwhile, the band at 2101 cm^-1^ decreased gradually, which may be due to the migration of CO molecular from metallic Au to the negatively charged Au*^x^*^−^ or the electronic structure change of Au nanoparticle. After treatment the Au/TiO_2_-H500 catalyst with O_2_ at 400 °C, the CO adsorption on negative change Au*^x^*^−^ disappeared and only the CO-Au^0^ was observed in the spectra. The infrared data indicate our Au/TiO_2_ catalyst does not exhibit the classical SMSI. The pretreatment with H_2_ at high temperature led to negatively charged Au after CO adsorption. This conclusion is further supported by the TEM study and catalytic test of CO oxidation.

The TEM images, as shown in Supplementary Figure 12, for both the Au/TiO_2_-H500 and Au/TiO_2_-H500-O400 samples, do not provide evidence for an encapsulating titania layer surrounding the gold nanoparticles.

One of the key features of classical SMSI is that the activity of the catalyst is reversible and strongly affected by the pretreat gas. The catalytic performance will be suppressed by the H_2_ pretreatment and restored through reoxidation by O_2_. But for our catalyst, the catalytic performance is completely different compared to the classical SMSI. Over two cycles of redox treatments, the CO conversion did not show strong differences (Supplementary Figure 13). The pretreatment with H_2_ in fact slightly enhanced catalyst activity, in contrast to the expected deactivation for SMSI. In view of the FTIR data, the enhancement of the activity can be due to the presence of negatively charged gold.

The above shows that the Au/TiO_2_ in this study does not possess the classical SMSI. It is in line with most studies revealing that SMSI is absent on Au/TiO_2_ except for a recent study that reported the classical SMSI for large-size gold nanoparticles (4.8-5.2 nm)^2^.


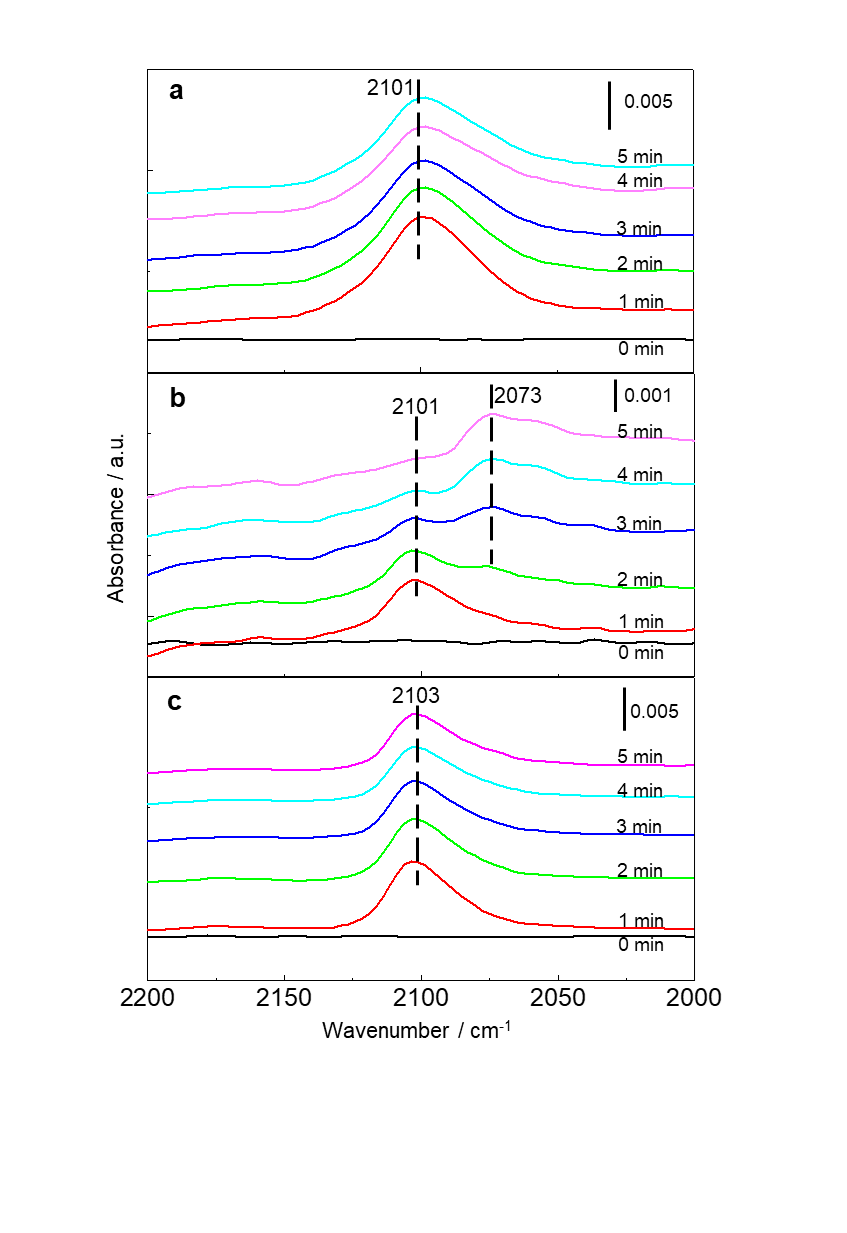


**Supplementary Figure 11.** **Sequence of in situ DRIFT spectra recorded during CO adsorption (1% CO in He) on various Au/TiO_2_ samples.** (**a**) Au/TiO_2_, (**b**) Au/TiO_2_-H500 (10 % H_2_/He at 500 °C) and (**c**) Au/TiO_2_-H500-O400 (10 % H_2_/He at 500 °C followed by 10 % O_2_/He at 400 °C). Experimental conditions: Flow rate is 30 ml min^-1^; feed gas is 1% CO diluted by He.


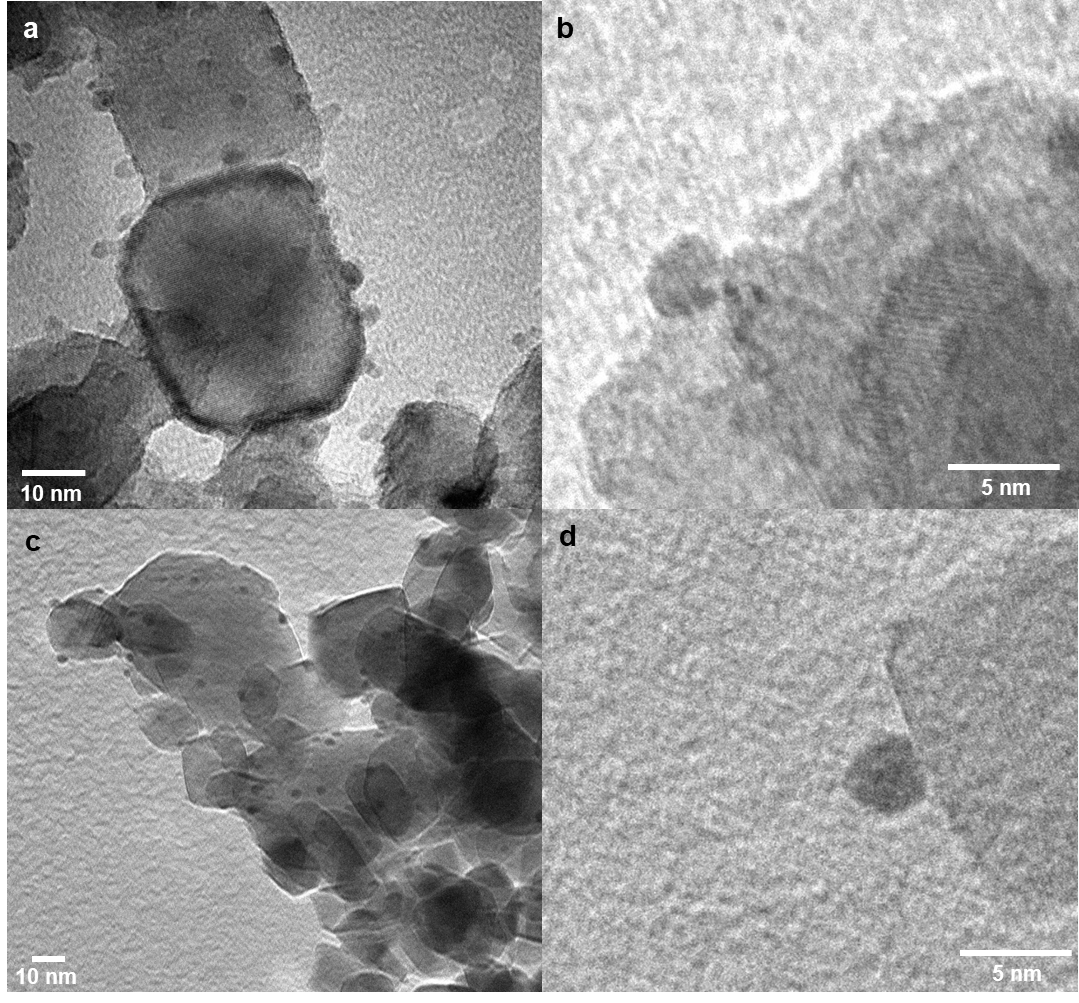


**Supplementary Figure 12.** **TEM image of the sample of Au/TiO_2_ with different pretreatment.** (**a**, **b**) Au/TiO_2_-H500, and (**c**, **d**) Au/TiO_2_-H500-O400.


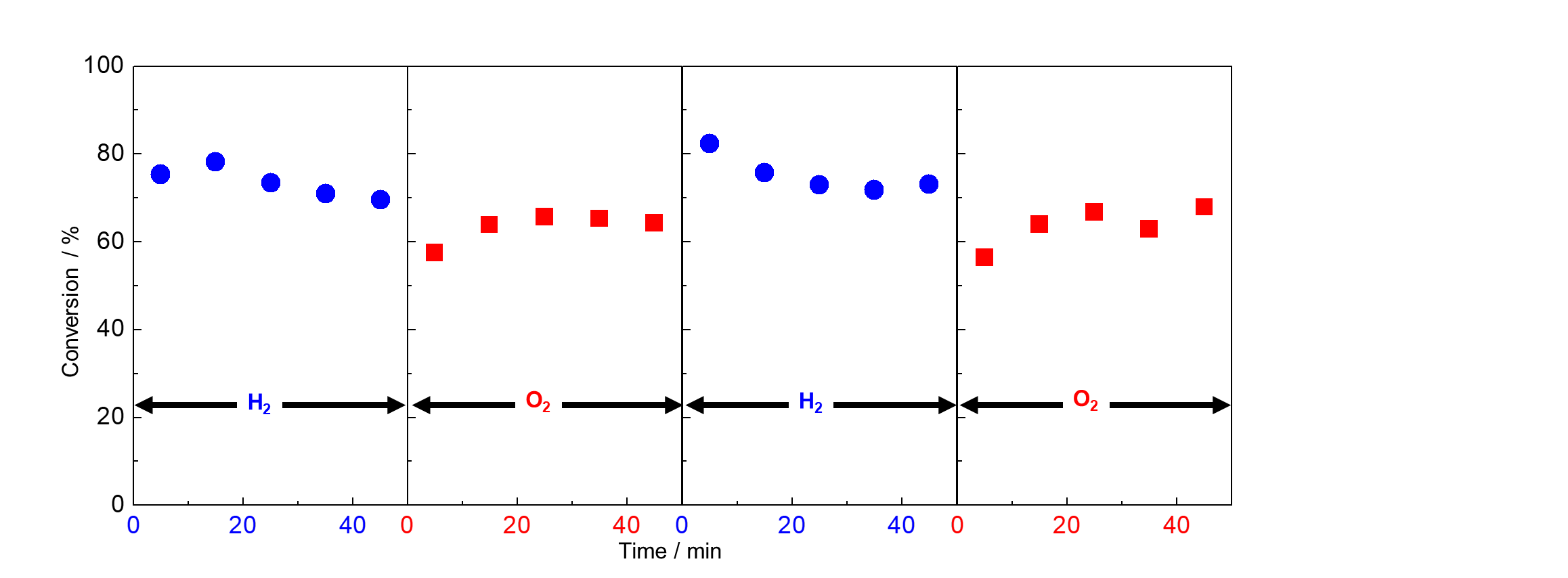


**Supplementary Figure 13.** **CO conversion at 60 °C for Au/TiO_2_ fresh sample following alternating pretreatment.**  Pretreated with 10 % H_2_/He at 500 °C (for the first and third cycles) or 10 % O_2_/He at 400 °C (for the second and fourth cycles). Reaction condition: 1% CO +1% O_2_ balanced with He; gas flow rate is 30 ml min^-1^; amount of sample is 30 mg.

**Water-gas-shift reaction over Au/TiO_2_**

The catalytic activity of water-gas-shift reaction was evaluated with a fixed bed reactor at atmospheric pressure. Typically, 100 mg of catalyst was loaded in the reactor. 30 ml min^-1^ 1% CO in He was first flowed throw liquid H_2_O, and then flowed throw the catalyst bed. The temperature was increased from 20 °C to 260 °C with a ramp rate of 5 °C min^-1^. A flow rate of 30 ml min^-1^ was used; feed gas is 1% CO in He saturated with H_2_O vapor; modulation period is 100 s; number of spectra in one cycle is 60; during the first half-cycle CO and H_2_O in He was flowed through the cell, then the feed gas was switched to He for the second half-cycle.

The activity of the Au/TiO_2_ in the water-gas-shift reaction is shown in Supplementary Figure 14. Au/TiO_2_ is very active in the water gas shift reaction. The CO conversion increased with the increase in temperature, and more than 90% of CO reacted with the water gas at 260 °C.

Then the DRIFTS MES was applied to study the mechanism of the water gas shift reaction on the Au/TiO_2_ surface. Supplementary Figure 15 shows the modulation spectra data during the reaction. A band between 2100 to 2114 cm^-1^, assigned to CO adsorption on Au (CO-Au), emerged in the spectra. The CO-Au band shows a slight blue shift at higher temperatures due to the low coverage of CO on the catalyst surface, because of the fast reaction rate at high temperatures. Another two bands at 2332 and 2363 cm^-1^ are assigned to CO_2_ were also observed for all temperatures, demonstrating that the catalyst is active. However, the transient inactivation effect is absent for all applied reaction conditions. It further confirms the proposal that the O_2_ also participates in the reconstruction of Au. The reconstruction of Au nanoparticles by CO and O_2_ leads to the transient inactivation effect.


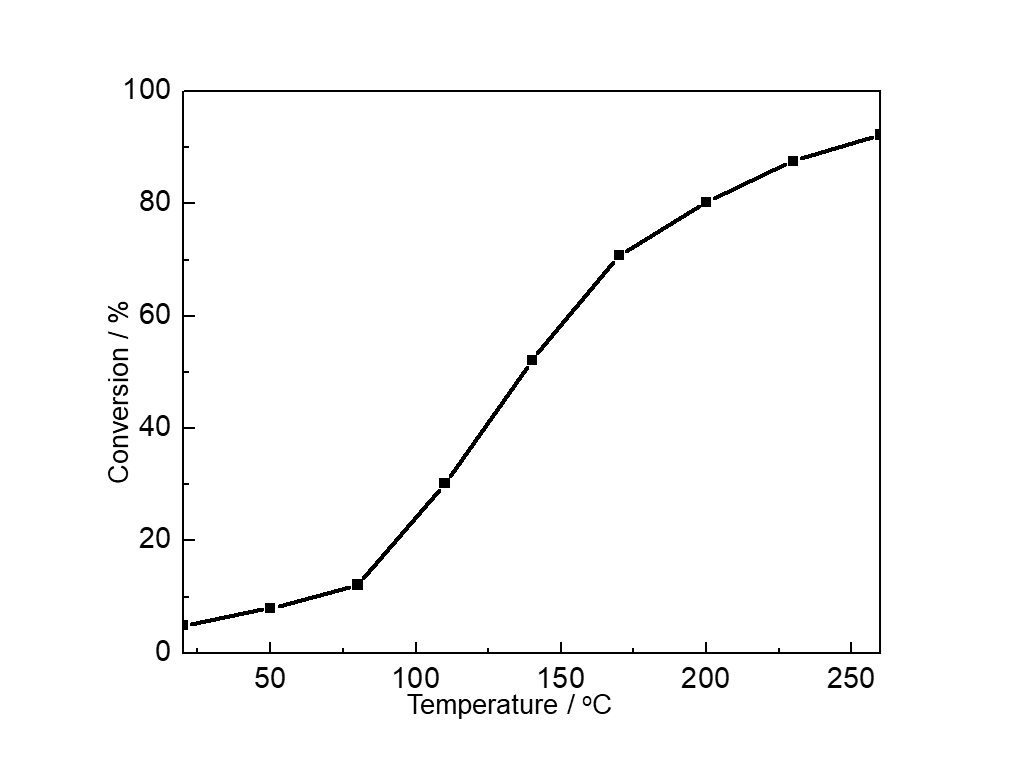


**Supplementary Figure 14.** **CO conversion as a function of reaction temperature over Au/TiO_2_ for water-gas-shift reaction.** Experimental conditions: Amount of sample is 100 mg; flow rate is 30 ml min^-1^. 1% CO in He was first bubbled throw liquid H_2_O, and then flowed throw the catalyst; the temperature was increased from 20 °C to 260 °C with a ramp rate of 5 °C min^-1^.


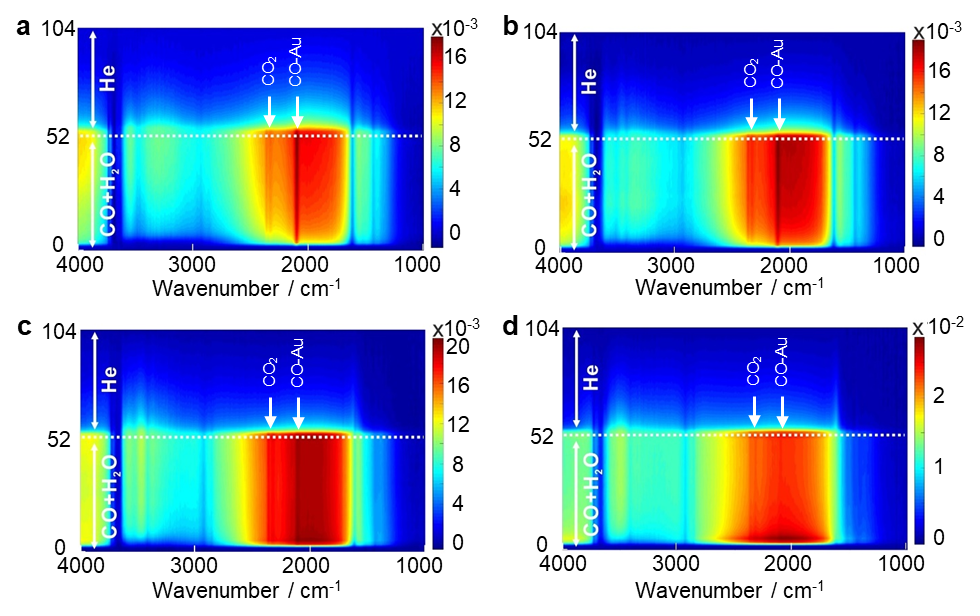


**Supplementary Figure 15.** **FTIR data of water-gas-shift reaction over Au/TiO_2_ at various temperatures.** (**a**) 100 °C, (**b**) 150 °C, (**c**) 200 °C and (**d**) 250 °C. Reaction conditions: flow rate is 30 ml min^-1^; feed gas is 1% CO in He which is firstly bubbled throw liquid H_2_O; modulation period is 100 s; number of spectra in one cycle is 60; during the first half-cycle CO and H_2_O in He was flowed through the cell, then the feed gas was switched to He for the second half-cycle.

**Supplementary References**

1. Green IX, Tang W, Neurock M, Yates JT, Jr. Spectroscopic observation of dual catalytic sites during oxidation of CO on a Au/TiO_2_ catalyst. *Science* **333**, 736-739 (2011).

2. Tang H*, et al.* Classical strong metal–support interactions between gold nanoparticles and titanium dioxide. *Sci. Adv.* **3**, e1700231 (2017).
